# Primate retinal cones express phosphorylated tau associated with neuronal degeneration yet survive in old age

**DOI:** 10.1016/j.exer.2017.09.013

**Published:** 2017-12

**Authors:** Asmaa Aboelnour, Jacqueline Van der Spuy, Michael Powner, Glen Jeffery

**Affiliations:** a Histology and Cytology Department, Faculty of Veterinary Medicine, Damanhour University, Egypt; b Institute of Ophthalmology, University College London, UK; c Department of Optometry and Visual Science, City University London, UK

**Keywords:** Retina, Aging, Mitochondria, Cone photoreceptor

## Abstract

Photoreceptor cells have high energy demands and suffer significantly with age. In aged rodents both rods and cones are lost, but in primates there is no evidence for aged cone loss, although their function declines. Here we ask if aged primate cones suffer from reduced function because of declining metabolic ability. Tau is a microtubule associated protein critical for mitochondrial function in neurons. Its phosphorylation is a feature of neuronal degeneration undermining respiration and mitochondrial dynamics. We show that total tau is widely distributed in the primate outer retina with little age-related change, being present in both rods and cones and their processes. However, all cones specifically accumulate phosphorylated tau, which was not seen in rods. The presence of this protein will likely undermine cone cell function. However, tau phosphorylation inhibits apoptosis. These data may explain why aged primate cones have reduced function but appear to be resistant to cell death. Consequently, therapies designed to remove phosphorylated tau may carry the risk of inducing cone photoreceptor cell death and further undermine ageing visual function.

## Highlights

• Primate cones are not lost with age, but decline in function.
• We show that primate cones specifically accumulate phosphorylated tau.
• Phosphorylated tau inhibits mitochondrial function, but protects from apoptosis.

Retinal function declines with age and this is associated with progressive inflammation and extra-cellular deposition including that on Bruch's membrane that restricts outer retinal metabolic supply. The consequence is progressive outer retinal cell loss (Bonnel et al., 2003, Xu et al., 2009, Pauleikhoff et al., 1990). In rodents there is early cone photoreceptor loss followed by reductions in rod photoreceptor numbers (Cunea and Jeffery, 2007, Cunea et al., 2014, Kolesnikov et al., 2010). But these patterns are not seen in humans and non-human primates. Here aged decline is apparent in both rod and cone mediated visual function (Birch and Anderson, 1992) and significant rod loss in the central retina is established by around 70 years of age. However, there is no evidence for age related cone loss not even among short wavelength sensitive cones that show the greatest functional vulnerability (Curcio et al., 1993; Weinrich et al., 2017). Even with the development of aged retinal disease, such as age-related macular degeneration, central cones show marked resistance to apoptosis even when the rod population around them has been lost due to geographic atrophy and their own function has declined (Curcio et al., 1996).

Photoreceptor cells have very high energy demands (Linsenmeier and Padnick-Silver, 2000). Their mitochondria that are a key intra-cellular energy source suffer functional decline resulting in reduced adenosine triphosphate (ATP) production, which is the currency of cellular function (Calaza et al., 2015). Within the ageing process, it is also known that mitochondria play a critical role in signaling cell death via the permeabilisation of their outer membrane, the release of cytochrome C and the initiation of caspase activation (Tait and Green, 2013). Tau is a microtubule protein critical for normal mitochondrial dynamics and whose phosphorylation is associated with reduced cellular function but also with protection from apoptosis (Wang and Mandelkow, 2016, Wang et al., 2010, Li et al., 2007). Here we show that primate cones specifically accumulate phosphorylated tau, potentially explaining their reduced function but also their survival in ageing.

The primate retinae used were from healthy young and old Macaca fascicularis from an established colony maintained by Public Health England (PHE). All animals were housed in compatible social groups, in accordance with the Home Office (UK) Code of Practice for the housing and Care of Animals Bred, Supplied or Used for Scientific Purposes, December 2014, and the National Committee for Refinement, Reduction and Replacement (NC3Rs), Guidelines on Primate Accommodation, Care and Use, August 2006 (NC3Rs, 2006). All animal procedures were approved by PHE Ethical Review Committee, Porton Down, UK, and authorised under an appropriate UK Home Office project license. Eyes were retrieved at death following sedation with ketamine and overdose of intra-venous sodium pentobarbital. The primary purpose of animal usage was different from the aims of this study. Eyes were removed and placed in 4% paraformaldehyde in phosphate buffer (PB) for approximately 24 h with only one eye being used for each animal. Following fixation, eyes were washed in PB. The anterior eye was removed and the retina and retinal pigmented epithelium were dissected free as a whole mount. These were dissected into defined regions and cryoprotected in 30% sucrose in PB and embedded in optimum cutting temperature compound (Agar Scientific Ltd) before sectioning in a cryostat at 10 μm. Sections were thaw mounted on slides and air dried and stored at −80C. Sections from 6 eyes were stained, 3 from 3 year old animals and 3 from 15 year old animals. Sections were then blocked in 5% NDS in PBST for 1 h then incubated overnight with mouse monoclonal to T-46 with dilution 1:1000 (1:1000. Thermoscientific, UK) for total tau or mouse anti tau (AT8; 1:100, Thermoscientific, UK) for phosphorylated tau. They were then washed and exposed to 0.3% H_2_O_2_ in PBS for 30 min to quench endogenous peroxidase. Sections were incubated with biotin-SP conjugated secondary antibodies; donkey anti mouse (1:1000, Jackson ImmunoResearch Laboratories, UK) diluted in 1% NDS in PBST. Sections were then incubated in horseradish peroxidase solution (Vector Laboratories, Peterborough, UK) for 30 min. Chromogenic visualization was achieved with 3,3-diaminobenzidine as peroxidase substrate by incubation for 1 min (Dako, USA). Antibody specificity was confirmed using Western blot. Tissues were washed and cover slipped with glycerol (100% graded molecules). Tissue was examined at a wide range of retinal locations, but here the data presented were from the macular region. Cones were clearly identified on the basis of their distinct morphology that separates them from rod photoreceptors, and this was apparent in all sections.

**Fig. 1:**
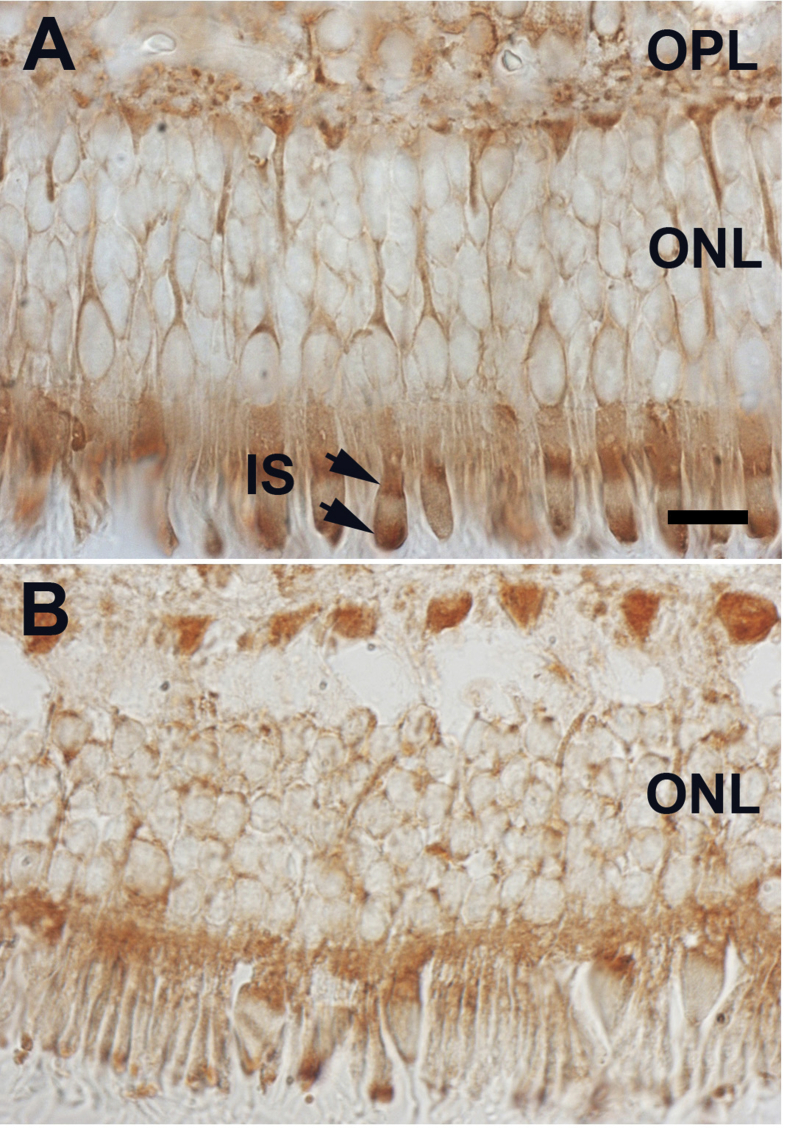
Patterns of labelling for total tau. A. in a 3 year old primate and B. in a 15 year old primate. In both label is present in rod and cone photoreceptors. It is also present in processes running through the ONL and in terminals in the OPL. In cone inner segments it was commonly present in two patches located at the proximal and distal regions, which are indicated by the arrows in A. No label was seen in outer segments. Tissue integrity in retinae in the older group was poorer than in the younger animals and this is reflected in the general quality of the histology between them. Abbreviations: Outer nuclear layer: ONL. Outer plexiform layer: OPL. Scale bar = 10 μm.

In all stained sections, irrespective of retinal location or age, total tau was present in both rods and cones including their inner segments and their processes running through the outer nuclear layer. In the photoreceptor cell inner segment, label was commonly present towards the base and at the tip, just under the outer segment. Label at the tip was clearly located in the ellipsoid region of the inner segment, however the location of the second band was relatively ambiguous. Associated label was also present in the outer plexiform layer. No staining was apparent in the outer segments or in the retinal pigmented epithelium or the choroid (Fig. 1). The patterns shown in the figure which were from the macular regions were representative of all retinal regions.

**Fig. 2:**
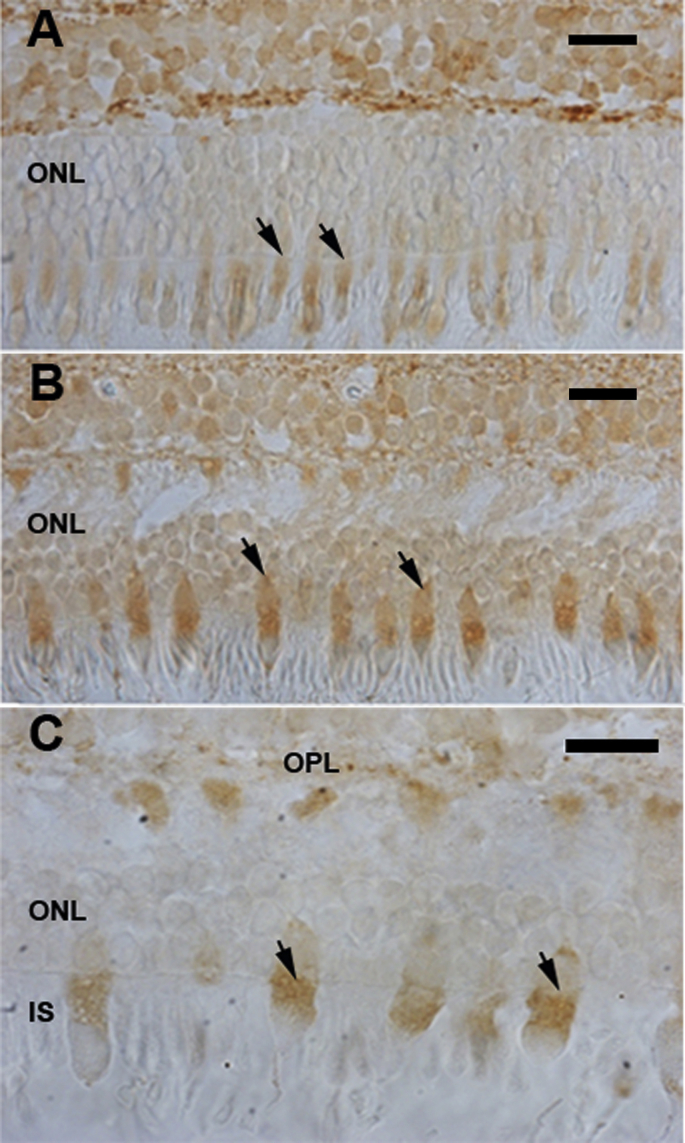
Patterns of phosphorylated tau staining in primate retina. A. phosphorylated tau in the outer retina of a 3 year old primate and B. phosphorylated tau in a 15 year old primate. Patterns of labelling are very similar in both retinae in that they are largely confined to inner segments of cones, but within this region label is heavier in the older animals. Staining is also present in the corresponding outer plexiform layer (OPL). There is also light staining in the cell body. C. Shows an aged primate retina at a higher magnification. Arrows in A, B and C provide examples of labelled cones. Abbreviations: Outer nuclear layer: ONL. IS: Inner segments. Scale bar A and B = 80 μm. C = 10 μm.

Patterns of staining for phosphorylated tau were very different. Fig. 2 shows a representative image of the outer retina from the macular region. However, staining patterns were similar in other regions of the outer retina. In every region where a cone was identified morphologically, phosphorylated tau was present in the inner segment. Further, in each case it accumulated predominantly in the myeloid region, although in many cones it also appeared to encroach upon the ellipsoid region. In each case there appeared to be more phosphorylated tau in aged primate cones compared to that in the younger animals (Fig. 2A and B). Within each group the density of label was similar between individual cones and across animals. This label was granular (Fig. 1C). However, patches of label were also present in the corresponding cone photoreceptor terminals in the outer plexiform layer. No such label was identified in structures similar to rods at any retinal location, nor was there any label in process running through the outer nuclear layer as seen for total tau. There were no obvious systematic changes in the distribution of phosphorylated tau in the inner retina. Attempts were made to quantify age related changes in phosphorylated tau in the outer retina using Western blot. However, it was not possible to target specifically the tissue of interest. Hence in whole retinal preparations changes in this signal were lost in the wider signal originating from other regions.

Tau is a microtubule associated protein that promotes microtubule polymerisation and stabilisation. Its hyperphosphorylation induces its detachment form microtubules, thereby destabilising them resulting in their depolymerisation. The hyperphosphorylation of tau in neurodegenerative disorders such as tauopathies, is associated with disruption of the neuronal microtubule tracks, which leads to deficits in mitochondrial mobility and synaptic docking (Trinczek et al., 1999, Stamer et al., 2002, Thies and Manddelkow, 2007, Dubey et al., 2008, LaPointe et al., 2009, Stoothoff et al., 2009, Kanaan et al., 2011, Kopeikina et al., 2011, Reddy, 2011, Shahpasand et al., 2012, Gilley et al., 2012). Mitochondrial function is also altered, with hyperphosphorylated tau specifically affecting mitochondrial bioenergetics (Pajak et al., 2016). But this can be a two way process as compromised mitochondrial function, particularly that of complex I can be associated with pathological changes in tau (Höglinger et al., 2005, Escobar-Khondiker et al., 2007).

In addition to the reduced mitochondrial synaptic docking, increased lipid peroxidation of the mitochondrial membrane is thought to alter neuronal bioenergetics and contribute to synaptic dysfunction. Interestingly, it has been shown that phosphorylated tau can directly insert into the planar lipid bilayer of the mitochondrial membrane to form ion-permeable channels and impair parkin-mediated mitophagy (DuBoff et al., 2012, Patel et al., 2015: Hu et al., 2016). Conversely, mitochondrial dysfunction and the resultant production of reactive oxygen species (ROS), by initiating membrane lipid peroxidation, can promote the phosphorylation of tau (Davis et al., 1997, Mattson et al., 1997, Zambrano et al., 2004, Galas et al., 2006, Melov et al., 2007, Iijima-Ando et al., 2012, Kandimalla et al., 2016). Thus, a vicious cycle between mitochondrial function and integrity and tau phosphorylation would ensue. The significance of the expression of hyperphosphorylated tau in photoreceptors during normal ageing and in the absence of photoreceptor degeneration is unclear, though it is possible it may be related to the high-energy demands of photoreceptors and a change in photoreceptor mitochondria-mediated bioenergetics with age. However, reduced cellular energy, increased ROS and changes in synaptic activity as seen in the cone end feet may all contribute to declining function.

Neurons in chronic neurodegenerative diseases like Alzheimer's accumulate hyperphosphorylated tau and contain neurofibrillary tangles of which hyperphosphorylated tau is a major component, but only a relatively small number of these cells die via apoptosis (Li et al., 2007, Jelling, 2001). Wang et al. (2010) and Li et al. (2007) have shown that hyperphosphorylated tau has the ability to antagonize apoptosis by stabilising β-catenin and increasing its nuclear translocation to promote cell survival. Moreover, Wang et al. (2010) reported that hyperphosphorylated tau reduced the release of cytochrome C from mitochondria as well as caspase-9 and caspase-3 activity, rendering cells more resistant to apoptosis. The data presented by Li et al. (2007) and Wang et al. (2010) are the likely explanation why cones survive for long periods, albeit in a relatively dysfunctional state. In light of these results, a cautious approach to the removal of hyperphosphorylated tau should be adopted if it comes at the price of increased probability of cone cell death. Further, these results highlight significant differences between mice and primates in patterns of retinal ageing that question the use of former as a model in the analysis of mechanisms in the aged human.

## References

[bib1] Birch D., Anderson J.L. (1992). Standardized full-field electroretinography: normal values and their variation with age. Arch. Ophthalmol..

[bib2] Bonnel S., Mohand-Said S., Sahel J.A. (2003). The aging retina. Exp. Gerontol..

[bib3] Calaza C., Kam J.H., Hogg C., Jeffery G. (2015). Mitochondrial decline precedes phenotype development in the complement factor H mouse model of retinal degeneration but can be corrected by near infrared light. Neurobiol. Aging.

[bib4] Cunea A., Jeffery G. (2007). The ageing photoreceptor. Vis. Neurosci.

[bib5] Cunea A., Powner M.B., Jeffery G. (2014). Death by color: differential cone loss in the aging mouse retina. Neurobiol. Aging.

[bib6] Curcio C.A., Millican C.L., Allen K.A., Kalina R.E. (1993). Aging of the human photoreceptor mosaic: evidence for selective vulnerability of rods in the central retina. Invest.Ophthalmol.Vis. Sci..

[bib7] Curcio C.A., Medeiros N.E., Millican C.L. (1996). Photoreceptor loss in age-related macular degeneration. Invest. Ophthalmol.Vis Sci..

[bib8] Davis D.R., Anderton B.H., Brion J.P., Reynolds C.H., Hanger D.P. (1997). Oxidative stress induces dephosphorylation of tau in rat brain primary neuronal cultures. J. Neurochem..

[bib9] Dubey M., Chaudury P., Kabiru H., Shea T.B. (2008). Tau inhibits anterograde axonal transport and perturbs stability in growing axonal neurites in part by displacing kinesin cargo: neurofilaments attenuate tau-mediated neurite instability. Cell Motil. Cytoskelet..

[bib10] DuBoff B., Gotz J., Feany M.B. (2012). Tau promotes neurodegeneration via DRP1 mislocalization in vivo. Neuron.

[bib11] Escobar-Khondiker M., Hollerhage M., Murie M.P., Champy P., Bach A., Depienne C., Respondek G., Yamada E.S., Lannuzel A., Yagi T., Hirsch E.C., Oertel W.H., Jacob R., Michel P.P., Ruberg M., Höglinger G.U. (2007). Annonacin, a natural mitochondrial complex I inhibitor, causes tau pathology in cultured neurons. J. Neurosci..

[bib12] Galas M.C., Dourlen P., Begard S., Ando K., Blum D., Hamdane M., Buée L. (2006). The peptidylprolyl cis/trans-isomerase Pin1 modulates stress-induced dephosphorylation of Tau in neurons: implication in a pathological mechanism related to Alzheimer disease. J. Biol. Chem..

[bib13] Gilley J., Seereeram A., Ando K., Mosely S., Adnrews S., Krechensteiner M., Misgeld T., Brion J.P., Anderton B., Hanger D.P., Coleman M.P. (2012). Age-dependent axonal transport and locomotor changes and tau hypophosphorylation in a “P301L” tau knockin mouse. Neurobiol. Aging.

[bib14] Höglinger G.U., Lannuzel A., Khondiker M.E., Mitchel P.P., Duyckaerts C., Feger J., Champy P., Prigent A., Medja F., Lombes A., Oertel W.H., Ruberg M., Hirsch E.C. (2005). The mitochondrial complex I inhibitor rotenone triggers a cerebral tauopathy. J. Neurochem..

[bib15] Hu Y., Li X.C., Wang Z.H., Luo Y., Zhang X., Liu X.P., Feng Q., Wang Q., Yue Z., Chen Z., Ye K., Wang J.Z., Liu G.P. (2016). Tau accumulation impairs mitophagy via increasing mitochondrial membrane potential and reducing mitochondrial Parkin. Oncotarget.

[bib16] Iijima-Ando K., Sekiya M., Maruko-Otake A., Ohtake Y., Suzuki E., Lu B., Iijima K.M. (2012). Loss of axonal mitochondria promotes tau-mediated neurodegeneration and Alzheimer's disease-related tau phosphorylation via PAR-1. PLoS Gent.

[bib17] Jelling K.A. (2001). Cell death in neurodegeneration. J.Cell. Mol. Med..

[bib18] Kanaan N.M., Morfini G., LaPointe N.E., Pigino G.F., Patterson K.R., Song Y., Andreadis A., Fu Y., Brady S.T., Binder L.I. (2011). Pathogenic forms of tau inhibit kinesin-dependent axonal transport through a mechanism involving activation of axonal phosphotransferases. J. Neurosci..

[bib19] Kandimalla R., Manczak M., Fry D., Suneetha Y., Sesaki H., Reddy P.H. (2016). Reduced dynamin-related protein 1 protects against phosphorylated tau-induces mitochondrial dysfunction and synaptic damage in Alzheimer's disease. Hum. Mol. Genet..

[bib20] Kolesnikov A.V., Fan J., Crouch R.K., Kefalov V.J. (2010). Age-related deterioration of rod vision in mice. J. Neurosci..

[bib21] Kopeikina K.J., Carlson G.A., Pitstick R., Ludvigson A.E., Peters A., Luebke J.I., Koffie R.M., Frosch M.P., Hyman B.T., Spires-Jones T.L. (2011). Tau accumulation causes mitochondrial distribution deficits in neurons in a mouse model of Tauopathy and in human Alzheimer's disease brain. Am. J. Pathol..

[bib22] LaPointe N.E., Morfini G., Pigino G., Gaisina I.N., Kozikowski A.P., Binder L.I., Brady S.T. (2009). The amino terminus of tau inhibits kinesin-dependent axonal trans- port: implications for filament toxicity. J. Neurosci. Re.

[bib23] Li H.L., Wang H.H., Liu S.J., Deng Y.Q., Zhang Y.J., Tian Q., Wang X.C., Chen X.Q., Yang Y., Zhang J.Y., Wang Q., Xu H., Liao F.F., Wang J.Z. (2007). Phosphorylation of tau antagonizes apoptosis by stabilizing beta-catenin, a mechanism involved in Alzheimer's neurodegeneration. Proc. Natl. Acad. Sci.U S A.

[bib24] Linsenmeier R.A., Padnick-Silver L. (2000). Metabolic dependence of photoreceptors on the choroid in the normal and detached retina. Invest.Ophthalmol.Vis. Sci..

[bib25] Mattson M.P., Fu W., Waeq G., Uchida K. (1997). 4-Hydroxynoneal, a product of lipid peroxidation, inhibits dephospohrylation of the microtubule-associated protein Tau. Neuroreport.

[bib26] Melov S., Adlard P.A., Morton K., Johnson F., Golden T.R., Hinerfeld D., Schilling B., Mavros C., Masters C.L., Volitakis I., Li Q.X., Laughton K., Hubbard A., Cherny R.A., Gibson B., Bush A.I. (2007). Mitochondrial oxidative stress causes hyperphosphorylation of tau. PLoS One.

[bib27] Pajak B., Kania E., Orzechowski A. (2016). Killing me softly: connotations to unfolded protein response and oxidative stress in Alzheimer's disease. Oxidative Med. Cell Longev..

[bib28] Patel N., Ramachandran S., Azimov R., Kagan B.L., Lal R. (2015). Ion channel formation by tau protein: implications for Alzheimer's disease and Tauopathies. Biochemistry.

[bib29] Pauleikhoff D., Harper C.A., Marshall J., Bird A. (1990). Aging changes in Bruch's membrane. A histochemical and morphological study. Ophthamol.

[bib30] Reddy P.H. (2011). Abnormal tau, mitochondrial dysfunction. Impaired axonal transport of mitochondria, and synaptic deprivation in Alzheimer's disease. Brain. Res..

[bib31] Shahpasand K., Uemura I., Saitto T., Asano T., Hata K., Shibata K., Toyoshima Y., Haseqawa M., Hisanaga S. (2012). Regulation of mitochondrial transport and inter-microtubule spacing by tau phosphorylation at the sites hyperphosphorylated in Alzheimer's disease. J. Neurosci..

[bib32] Stamer K., Vogel R., Thies E., Mandelkow E., Mandelkow E.M. (2002). Tau blocks traffic of organelles, neurofilaments, and APP vesicles in neurons and enhances oxidative stress. J. Cell Biol..

[bib33] Stoothoff W., Jones P.B., Spires-Jones T.L., Joyner D., Chhabra E., Bercury K., Fan Z., Xie H., Bacskai B., Edd J., Irimia D., Hyman B.T. (2009). Differential effect of three-repeat and four-repeat tau on mitochondrial axonal transport. J. Neurochem..

[bib34] Tait S.W.G., Green D.R. (2013). Mitochondrial regulation of cell death. Cold Spring Harb. Perspect.Biol.

[bib35] Thies E., Manddelkow E.M. (2007). Missorting of tau in neurons causes degeneration of synapses that can be rescued by the kinase MARK2/Par-1. J. Neurosci..

[bib36] Trinczek B., Ebneth A., Mandelkow E.M., Mandelkow E. (1999). Tau regulated the attachment/detachment but not the speed of motors in microtubule-dependent transport of single vesicles and organelles. J. Cell. Sci..

[bib37] Wang Y., Mandelkow E. (2016). Tau in physiology and pathology. Nat. Revs. Neurosci..

[bib38] Wang H.H., Li H.L., Liu R., Zhang Y., Liao K., Wang J.Z., Liu S.J. (2010). Tau overexpression inhibits cell apoptosis with the mechanisms involving multiple viability-related factors. J. Alzheimers Dis..

[bib39] Weinrich T.W., Powner M.B., Lynch A., Jonnal R.S., Werner J.S., Jeffery G. (2017). No evidence for loss of short-wavelength sensitive cone photoreceptors in normal ageing of the primate retina. Sci. Reps.

[bib40] Xu H., Chen M., Forrester J.V. (2009). Para-inflammation in the aging retina. Prog. Ret. Eye Res..

[bib41] Zambrano C.A., Egana J.T., Nunez M.T., Maccioni R.B., González-Billault C. (2004). Oxidative stress promotes tau dephosphorylation in neuronal cells: the roles of cdk5 and PP1. Free Rad. Biol. Med..

